# Integrin triplets of marine sponges in the murine and human MHCI-CD8 interface and in the interface of human neural receptor heteromers and subunits

**DOI:** 10.1186/2193-1801-2-128

**Published:** 2013-03-22

**Authors:** Alexander O Tarakanov, Kjell G Fuxe

**Affiliations:** 1 Russian Academy of Sciences, St. Petersburg Institute for Informatics and Automation, Saint Petersburg, Russia; 2 Department of Neuroscience, Karolinska Institutet, Stockholm, Sweden

**Keywords:** Neural receptor-receptor interactions, Receptor interface, Marine sponges, Triplet homologies

## Abstract

Based on our theory, main triplets of amino acid residues have been discovered in cell-adhesion receptors (integrins) of marine sponges, which participate as homologies in the interface between two major immune molecules, MHC class I (MHCI) and CD8αβ. They appear as homologies also in several human neural receptor heteromers and subunits. The obtained results probably mean that neural and immune receptors also utilize these structural integrin triplets to form heteromers and ion channels, which are required for a tuned and integrated intracellular and intercellular communication and a communication between cells and the extracellular matrix with an origin in sponges, the oldest multicellular animals.

## Introduction

Based on a mathematical approach, Tarakanov and Fuxe (2010, 2011) have deduced a set of triplet homologies (so called ‘triplet puzzle’) that may be responsible for protein-protein interactions, including receptor heteromers and human immunodeficiency virus (HIV) entry. For example, the triplet of amino acid residues ITL (Ile-Thr-Leu) appears in both receptors of any of six receptor heteromers: GABAB1-GABAB2 (GABAB receptor), GABAB1-mGluR1, GABAB1-CXCR4, CXCR4-CCR2, 5HT1B-5HT1D, and MHC class I MHCI-CD8. At the same time, this triplet ITL does not appear in both receptors of any of known non-heteromers (GABAB2-A2A, A2A-D1, A1-D2, NTSR1-D1, TSHR-D2, and CD4-D2; see Tarakanov and Fuxe 2010). According to recent biochemical studies (Borroto-Escuela et al. 2010, 2011, [Bibr CR5_182][Bibr CR6_182]; Romero-Fernandez et al. 2011), such triplets exist in the interacting domains forming the receptor interface. Furthermore, a ‘guide-and-clasp’ manner of receptor-receptor interactions has been proposed where the ‘adhesive guides’ may be the triplet homologies (Tarakanov and Fuxe, 2010). According to recent bioinformatic studies (Tarakanov et al. [Bibr CR22_182][Bibr CR23_182][Bibr CR24_182][Bibr CR25_182]), several triplet homologies of such receptor heteromers in human brain may be the same as in cell-adhesion receptors of marine sponges, known to be highly conserved from the lowest metazoa to vertebrates (Gamulin et al. 1994; Muller 1997; Pancer et al. 1997; Buljan and Bateman 2009). Interactions between such triplets probably represent a general molecular mechanism for receptor-receptor interactions (Fuxe et al 2012) and may play an important role in human learning (Agnati et al. 2003) and some diseases (Tarakanov et al. 2009).

In the current paper, many of such triplets have been found in integrins of marine sponges together with human alpha and beta integrins. This means that such triplet homologies may play a role in alpha-beta heterodimeric complexes forming integrin receptors and interact with extracellular matrix proteins (Barczyk et al. 2010). Of especial interest is that the same integrin triplets exist also in the murine and human MHCI interface with CD8, in human neural receptors and in the interface of both protomers of several receptor heteromers. The presence of such triplet homologies in several receptor subunits building up the neuromuscular nicotinic cholinergic receptors has also been demonstrated. At least one of the homologies may have a role in the intermolecular subunit interactions of this ion channel receptor.

## Methods

Amino acid codes of receptors and other proteins have been obtained from the National Center for Biotechnology Information (http://www.ncbi.nlm.nih.gov) and the Universal Protein Resource (http://www.uniprot.org). Table 1 summarizes data on proteins used. In abstract mathematical terms, any protein is just a word coded by a 20-letter alphabet where triplet is any 3-letter subword. Thus, triplet homology is any triplet which exists in both given words. Our theory of triplet puzzle supposes some basic set of triplets as a code that determines whether two receptors bind or not (Tarakanov and Fuxe 2010). None of the widely used software like Clustal (http://www.clustal.org/), AGGRESCAN (http://bioinf.uab.es/aggrescan/), accelrys (http://accelrys.com/), and so on seems to be able to deal with so specific and complicated combinatorial puzzle. Our original software has been developed to determine such basic set of triplet homologies from two given sets of protein-protein pairs (which bind and do not bind). The core of this software is the computing of all triplet homologies between two given words (but not only their alignment like in the above mentioned Clustal). The method consists in forming the binary matrix of all one-letter homologies (which element is 1 if there is homology and 0 otherwise) and then filtering this matrix using rather specific rules of so called cellular automata (for example, see Tarakanov and Prokaev 2007; http://youtu.be/1DevThU5fyM).

**Table 1:** **Data on proteins used**

Protein	Species	Type	Accession code
ITGA	Sponge (*Geodia cydonium*)	Metazoan adhesion receptor subunit Integrin-α	CAA65943
ITGB	Sponge (*Geodia cydonium*)	Metazoan adhesion receptor subunit Integrin-β	CAA77071
ITGB4	Sponge (*Marichromatium purpuratum*)	Metazoan adhesion receptor subunit Integrin-β4	ZP_08774040
MHCI	Mouse (*Mus musculus*)	H-2 class I histocompatibility antigen	NP_001001892
CD8a	Mouse	T-cell surface glycoprotein chain CD8α	NP_001074579
CD8b	Mouse	T-cell surface glycoprotein chain CD8β	NP_033988
MHCI	Human (*Homo sapiens*)	H-2 class I histocompatibility antigen	AAA59599
CD8a	Human	T-cell surface glycoprotein chain CD8α	NP_001139345
CD8b	Human	T-cell surface glycoprotein chain CD8β	NP_757362)
CXCR4	Human	Chemokine receptor	P61073
TSHR	Human	Thyroid stimulating hormone receptor	NP_000360
FGFR1	Human	Fibroblast growth factor receptor	NP_075598
5HT1A	Human	Serotonin receptor	AAH69159
Collagen	Human	Matrix protein	P02452
ITGAIIB	Human	Integrin receptor subunit-αIIb	P08514
ITGAL	Human	Integrin receptor subunit-αL	P20701
ITGAM	Human	Integrin receptor subunit-αM	NP_001139280
ITGAV	Human	Integrin receptor subunit-αV	EAX10934
ITGAX	Human	Integrin receptor subunit-αX	NP_000878
ITGB2	Human	Integrin receptor subunit-β2	NP_000202
ITGB3	Human	Integrin receptor subunit-β3	NP_000203
ITGB4	Human	Integrin receptor subunit-β4	NP_000204
ITGB5	Human	Integrin receptor subunit-β5	NP_000205
ITGB6	Human	Integrin receptor subunit-β6	P18564
ITGB8	Human	Integrin receptor subunit-β8	P26012
ACHA	Human	Acetylcholine receptor subunit-α	P02708
ACHB	Human	Acetylcholine receptor subunit-β	P11230
ACHD	Human	Acetylcholine receptor subunit-δ	Q07001
ACHE	Human	Acetylcholine receptor subunit-ε	Q04844
mGluR1	Human	Metabotropic glutamate receptor	NP_000829
GABAB2	Human	γ-aminobutyric acid receptor subunit-2	O75899
GABAB1	Human (*Homo sapiens*)	γ-aminobutyric acid receptor subunit-1	NP_001461
GABAB1	Mouse (*Mus musculus*)	"	NP_062312
GABAB1	Norway rat (*Rattus norvegicus*)	"	NP_112290
GABAB1	Western clawed frog (*Xenopus (Silurana) tropicalis*)	"	NP_001107291
GABAB1	Green puffer (*Tetraodon nigroviridis*)	"	uniprot/Q4S9D9
GABAB1	Zebrafish (*Danio rerio*)	"	NP_001070794
GABAB1	African malaria mosquito (*Anopheles gambiae*)	"	uniprot/Q7PME5
GABAB1	*Drosophila pseudoobscura*	"	XP_001357356
GABAB1	Human body louse (*Pediculus humanus corporis*)	"	XP_002430445
GABAB1	*Caenorhabditis elegans*	"	ACE63490

**Table 2:** **Example of integrin triplets of marine sponges in murine and human proteins**

Protein	Species	Type	LLG	GLL	ITL	RPA	GDR	RDG	DGR
ITGA	Sponge	Integrin-α	-	-	+	+	+	-	-
ITGB	Sponge	Integrin-β	+	+	-	-	-	-	-
ITGB4	Sponge	Integrin-β	-	-	-	-	-	+	+
MHC Class I	Mouse	Immune receptor	+	-	+	+	-	-	+
CD8a	Mouse	Immune receptor	+	-	+	-	-	-	-
CD8b	Mouse	Immune receptor	-	-	-	-	-	-	-
MHC Class I	Human	Immune receptor	+	-	+	+	-	+	+
CD8a	Human	Immune receptor	-	-	+	+	-	-	-
CD8b	Human	Immune receptor	-	+	+	-	-	-	-
CXCR4	Human	Immune receptor	-	-	+	-	-	-	-
TSHR	Human	Endocrine receptor	-	-	-	+	-	-	-
FGFR1	Human	Receptor tyrosine kinase	-	-	-	+	-	-	-
5HT1A	Human	Neural receptor	+	-	-	-	-	-	-
Collagen	Human	Matrix protein	-	-	-	-	+	+	+
ITGAIIB	Human	Integrin-α	+	+	-	-	-	+	+
ITGAL	Human	Integrin-α	-	+	-	-	-	-	-
ITGAM	Human	Integrin-α	+	+	-	-	-	-	-
ITGAV	Human	Integrin-α	+	+	-	-	-	-	-
ITGAX	Human	Integrin-α	+	+	+	-	+		-
ITGB2	Human	Integrin-β	-	+	-	-	-	-	+
ITGB3	Human	Integrin-β	-	+	-	-	-	-	+
ITGB4	Human	Integrin-β	+	-	-	-	-	-	-
ITGB5	Human	Integrin-β	+	-	-	-	-	+	-
ITGB6	Human	Integrin-β	-	+	-	-	-	-	-
ITGB8	Human	Integrin-β	-	+	-	-	+	-	-
ACHA	Human	Neural receptor subunit	+	-	-	-	-	-	-
ACHB	Human	Neural receptor subunit	+	-	+	+	+	-	-
ACHD	Human	Neural receptor subunit	-	+	+	+	-	-	-
ACHE	Human	Neural receptor subunit	+	+	-	-	-	-	-
GABAB1	Human	Neural receptor	+	+	+	-	-	-	-
GABAB2	Human	Neural receptor	-	+	+	-	-	-	-
mGluR1	Human	Neural receptor	-	+	+	-	-	-	-

(+ yes, - no).

**Table 3:** **Example of integrin triplets of marine sponges in the protomers of human receptor heteromers and in subunits of the neuromuscular nicotinic receptor**

Receptor heteromer	Reference	Function	LLG	GLL	ITL	RPA	DGR
MHCI-CD8a	Gao et al. (1997)	Adaptive immune response	-	-	#	+	-
	Wang et al. (2009)						
MHC1-CD8b	Wang et al. (2009)	Adaptive immune response	-	-	#	-	-
CD8a-CD8b	Wang et al. (2009)	Coreceptor of T cells	-	-	+	-	-
ITGAIIB-ITGB3	Barczyk et al. (2010)	RGD (Arg-Gly-Asp) receptor	-	#	-	-	#
ITGAV-ITGB3	Barczyk et al. (2010)	RGD receptor	-	#	-	-	-
ITGAV-ITGB5	Barczyk et al. (2010)	RGD receptor	#	-	-	-	-
ITGAV-ITGB6	Barczyk et al. (2010)	RGD receptor	-	#	-	-	-
ITGAV-ITGB8	Barczyk et al. (2010)	RGD receptor	-	#	-	-	-
ITGAL-ITGB2	Barczyk et al. (2010)	Leukocyte receptor	-	+	-	-	-
ITGAM-ITGB2	Barczyk et al. (2010)	Leukocyte receptor	-	+	-	-	-
ITGAX-ITGB2	Barczyk et al. (2010)	Leukocyte receptor	-	+	-	-	-
GABAB1-GABAB2	Marshall et al. (2001)	Activation of the potassium channels and regulation of receptor trafficking	-	#	#	-	-
GABAB1-mGluR1	Hirono et al. (2001)	Modulation of excitatory transmission	-	#	+	-	-
GABAB1-CXCR4	Guyon and Nahon (2007)	Modulation of neuroendocrine systems	-	-	#	-	-
ACHA-ACHB	Changeux et al. 1984	Part of the neuromuscular nicotinic receptor	+	-	-	-	-
ACHA-ACHE	Changeux et al. 1984	Part of the neuromuscular nicotinic receptor	+	-	-	-	-
ACHB-ACHD	Changeux et al. 1984	Part of the neuromuscular nicotinic receptor	-	-	+	#	-

(+ yes in both receptors, # may mediate their interaction, - no in any receptor).

No experimental research has been performed on humans and/or animals.

## Results

**Figure 1:**
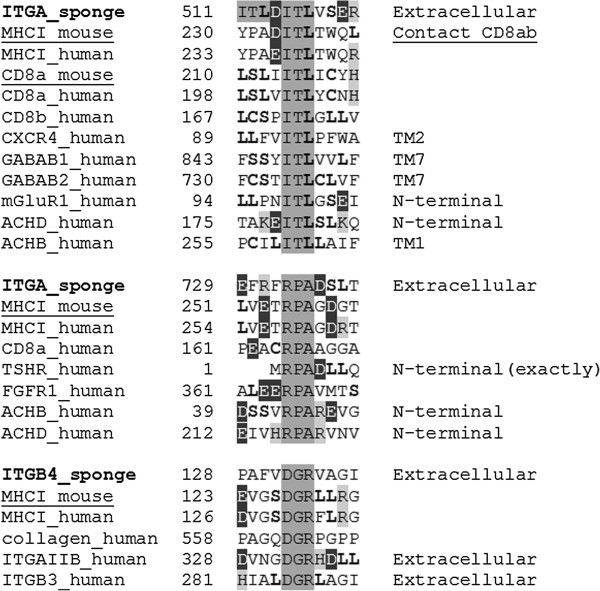
**Example of the triplets ITL, RPA, and DGR (dark-shaded letters) in the integrins of marine sponges existing in the murine (underlined) and human MHCI-CD8 complex, human collagen (DGR triplet), and human receptor heteromers: TM1, TM2 and TM7 are the first, the second and the seventh transmembrane α-helices of ACHB, CXCR4, and GABAB (GABAB1-GABAB2 heteromer) receptors, respectively, and contain the ITL triplet.** The RPA triplet is also found in the TSHR and FGFR1; the RPA but not the ITL triplet homologies are in a position to contribute to the physical interaction between the beta and delta subunits of the neuromuscular nicotinic receptor (ACHB-ACHD); light-shaded letters are positively charged amino acids (R, K, and H), whereas dark-shaded white letters are negatively charged amino acids (D and E); bold letters are main players of leucine-rich motifs (L, S, and C).

**Figure 2:**
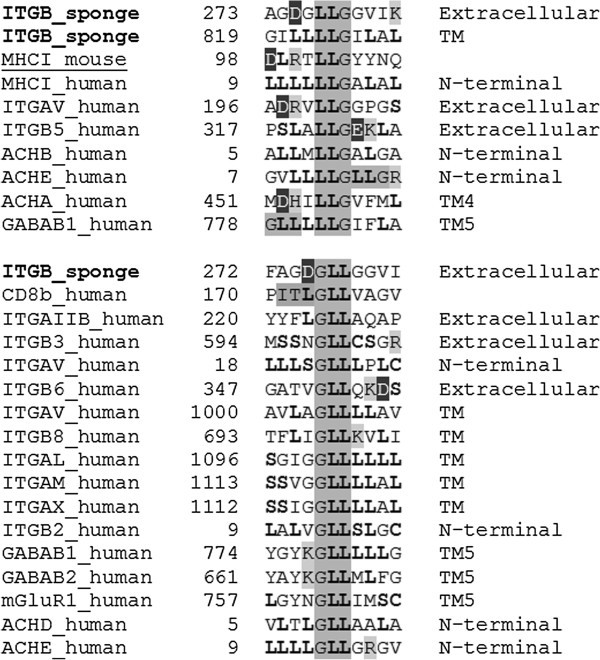
**Example of the triplets LLG and GLL (dark-shaded letters) in the integrins of marine sponges, murine (underlined) and human MHC Class I and human receptor heteromers.**

The triplets ITL (Ile-Thr-Leu), RPA (Arg-Pro-Ala), DGR (Asp-Gly-Arg), LLG (Leu-Leu-Gly), and GLL (Gly-Leu-Leu) of the integrin receptors of marine sponges appear as homologies in murine and human MHCI, GABAB1, and human integrin receptor heteromers (see Tables 2 and 3, Figures 1 and 2). The triplets ITL (Ile-Thr-Leu) and DGR (Asp-Gly-Arg) are particularly interesting. For example, the triplet ITL is in the interface providing the binding between MHCI and CD8αβ (Wang et al. 2009). This triplet homology exists also in three GABAB1 receptor heteromers of human brain: GABAB1-GABAB2 forming the GABAB receptor (Marshall et al. 2001), GABAB1-mGluR1, and GABAB1-CXCR4 and may mediate the interaction in two of them (see Table 3 and Figure 1). In the first two heteromers also triplet GLL (Gly-Leu-Leu) may participate in the interaction (see Table 3 and Figure 2).

The triplet DGR (Asp-Gly-Arg) is in fact the inverse triplet of RGD (Arg-Gly-Asp) that provides the binding site for integrin RGD-binding receptors (see Table 3). Moreover, a small peptide ligand RGD (Arg-Gly-Asp) that mimics extracellular matrix protein binding to integrins also causes impairments in plasticity at glutamatergic synapses (Wiggins et al. 2011).

**Figure 3:**
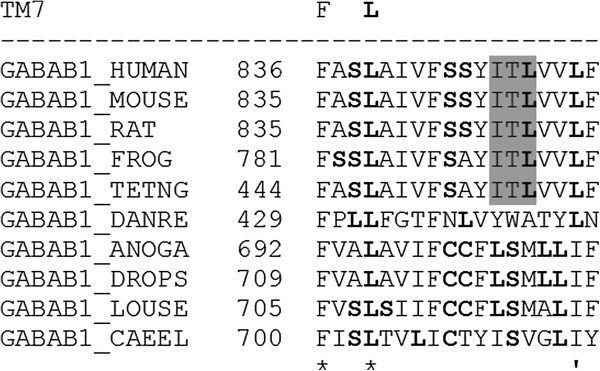
**The triplet ITL (dark-shaded letters) during the evolution of GABAB1 subunit: CAEEL** (***Caenorhabditis elegans***)**, LOUSE** (***Pediculus humanus corporis***)**, DROPS** (***Drosophila pseudoobscura***)**, ANOGA** (***Anopheles gambiae***)**, DANRE** (***Danio rerio***)**, TETNG** (***Tetraodon nigroviridis***)**, FROG** (***Xenopus tropicalis***)**, RAT** (***Rattus norvegicus***)**, MOUSE** (***Mus musculus***)**, and HUNAN** (***Homo sapiens***); asterisk (*) marks homologies (F and L); quote (') marks leucine-like homologies (L and I); bold letters are main players of leucine-rich motifs (L, S, and C).

The evolution of the ITL triplet in the GABAB1 receptor subunit is displayed in Figure 3. In phylogeny, it appears to begin in fish (*Tetraodon*) and then continues to man, while it is missing in zebrafish (*Danio rerio*). Thus, the usefulness of the ITL triplet in recognition is rediscovered in the fish GABAB1 receptor.

Furthermore, the RPA triplet homology in the beta and delta interacting nicotinic subunits of the neuromuscular nicotinic receptor (see Changeux et al. 1984) is in a location (N-terminal parts of ACHB and ACHD) where it may participate in forming part of their interface (see Figure 1 and Table 3).

## Discussion

The triplet ITL (Ile-Thr-Leu) found in integrins of marine sponges is presented as a homology in the interface between MHC Class I and CD8αβ heterodimer (coreceptor in T cells). It is postulated that this triplet homology can contribute to the formation of the MHCI-CD8 heteromeric complex which leads to a strong activation of the T cell by guiding the T-cell receptor into relevant self-MHC recognition (see Wang et al. 2009). Thus, it seems possible that the ITL triplet may have a critical role in the interaction between these two immune receptors which is necessary for appropriate T cell function. A mutation of the ITL triplet in these immune receptors will be of value to test this hypothesis. The indications have also been obtained that triplet homology ITL in the N-terminal of beta and delta nicotinic receptor subunits of the neuromuscular nicotinic receptor may help mediate their interaction in the subunit interface.

## Conclusion

Integrin triplets of marine sponges found in the interface of human receptor heteromers and even in the interface between two major immune molecules MHCI-CD8 seem to confirm once more our theory. This triplet puzzle arose as a surprising merger of pure mathematics and most recent biochemical studies of receptor-receptor interactions. As a result, it appears that neural and immune receptor heteromers in humans may also utilize these structural elements originating in sponges, the oldest multicellular animals. Thus, the triplet puzzle may be an ancient and general mechanism for protein-protein recognition.
